# Development and validation of a prognostic prediction model for patients with lung metastasis of esophageal cancer

**DOI:** 10.1186/s12957-025-04025-4

**Published:** 2025-10-14

**Authors:** Xianzhe Si, Lei Gao, Yifang Zhu, Jianan Chen, Zhiyao Chen, Xiaoli Chen

**Affiliations:** 1 Department of Gastrointestinal, Hernia and Abdominal Wall Surgery, Zhengzhou Central Hospital Affiliated to Zhengzhou University, Zhengzhou, 450000 Henan Province China; 2 Sanquan College of Xinxiang Medical University, Xinxiang, 453513 Henan Province China; 3 Department of Clinical Sciences, H. Lee Moffitt Cancer Center & Research Institute, 12902 USF Magnolia Drive, Tampa, FL 33612 USA; 4 Department of Gastrointestinal & Esophageal Surgery, the 2nd Affiliated Hospital of Fujian Medical University, Quanzhou, 362000 Fujian Province China; 5 Department of Nephrology, Quanzhou Traditional Chinese Medicine Ho spital, Quanzhou, 362000 Fujian Province China

**Keywords:** Esophageal cancer, Prediction model, Column chart, Survival prediction

## Abstract

**Purpose:**

Pulmonary metastasis is relatively rare among esophageal cancer patients, and there is a limited body of research in this regard. This study used clinical and pathological indicators from monitoring, epidemiological, and Surveillance, Epidemiology, and End Results (SEER) databases to look at the risk factors for patients who develop pulmonary metastasis. This study aims to explore the risk factors for lung metastasis in esophageal cancer patients and their impact on prognosis, and to construct a nomogram model for predicting the survival period of patients with lung metastasis.

**Methods:**

We obtained data on esophageal cancer patients from the Surveillance, SEER database between 2010 and 2015. Patients with esophageal cancer lung metastasis were divided into a training group and a testing group. Univariate and multivariate analyses were performed on the training set to identify independent risk factors for esophageal cancer lung metastasis, and a nomogram model was constructed to predict overall survival (OS). The model’s discriminatory ability and calibration were evaluated using the C-index, area under the receiver operating characteristic (ROC) curve (AUC), and calibration curve.

**Results:**

A total of 10,035 esophageal cancer patients were included in this study, with 590 diagnosed with lung metastasis. Single-factor and multi-factor logistic regression analyses revealed several risk factors associated with esophageal cancer lung metastasis, including male gender, higher tumor grades (Grade II and Grade III), advanced T stage, and the presence of bone, brain, and liver metastases. Patients diagnosed with esophageal cancer who have pulmonary metastases have a median overall survival of six months. For patients with esophageal cancer with lung metastasis, significant prognostic factors identified by Cox regression analysis included tumor grade (grade II and III), T2 lymph node metastasis, T3 lymph node metastasis, and the presence of bone metastasis. The constructed nomogram was validated by ROC analysis and calibration curves, demonstrating good predictive accuracy and discriminatory power.

**Conclusions:**

There are certain differences in risk and prognostic factors between patients with esophageal cancer and lung metastasis. ROC curves and calibration curves validated that the dynamic nomogram has certain predictive performance.

## Introduction

Esophageal cancer is a malignant tumor originating from the mucosal epithelium of the esophagus and one of the most common forms of cancer with highly aggressive clinical features and biological behavior [1]. As one of the seven most common cancers in the world, it ranks among the top six in mortality [2]– [3]. In recent years, the incidence of esophageal cancer, particularly the adenocarcinoma subtype, has been increasing in Western countries [4–6]. Epidemiologic data show that more than 30% of patients have metastases at the time of initial diagnosis [7–9]. While clinically, metastasis in esophageal cancer patients often occurs in nearby lymph nodes or distant organs, nearly half of the patients with metastatic esophageal cancer experience metastasis in locations such as the liver, brain, lungs, and bones [10–12]. Currently, no effective method for treating distant metastases of esophageal cancer has been found, and the available studies only indicate that surgical resection of lung metastases may benefit some patients [13]– [14], but the long-term benefits and associated costs of surgery are still issues to be resolved [15]– [16]. The treatment of esophageal cancer lung metastasis is tricky, and once lung metastasis occurs, it means that the disease has progressed to an advanced stage; therefore, it is of great significance to study the risk factors and prognostic factors of esophageal cancer lung metastasis. Therefore, developing a predictive model for metastatic esophageal cancer based on clinical and pathological indicators becomes particularly important.

The primary objective of this study was to construct and validate a dynamic nomogram for predicting the survival rate of esophageal cancer patients with lung metastasis based on data from the US Surveillance, SEER database from 2010 to 2015. We first used logistic regression analysis to identify risk factors associated with lung metastasis in the entire cohort of esophageal cancer patients. Subsequently, for patients with lung metastasis, we employed Cox regression analysis to explore independent prognostic factors for overall survival. Finally, we integrated these prognostic factors to establish a dynamic nomogram model and comprehensively validated its predictive performance using ROC curves and calibration curves in both the training and validation sets.

## Methods

### Data collection

**Fig. 1:**
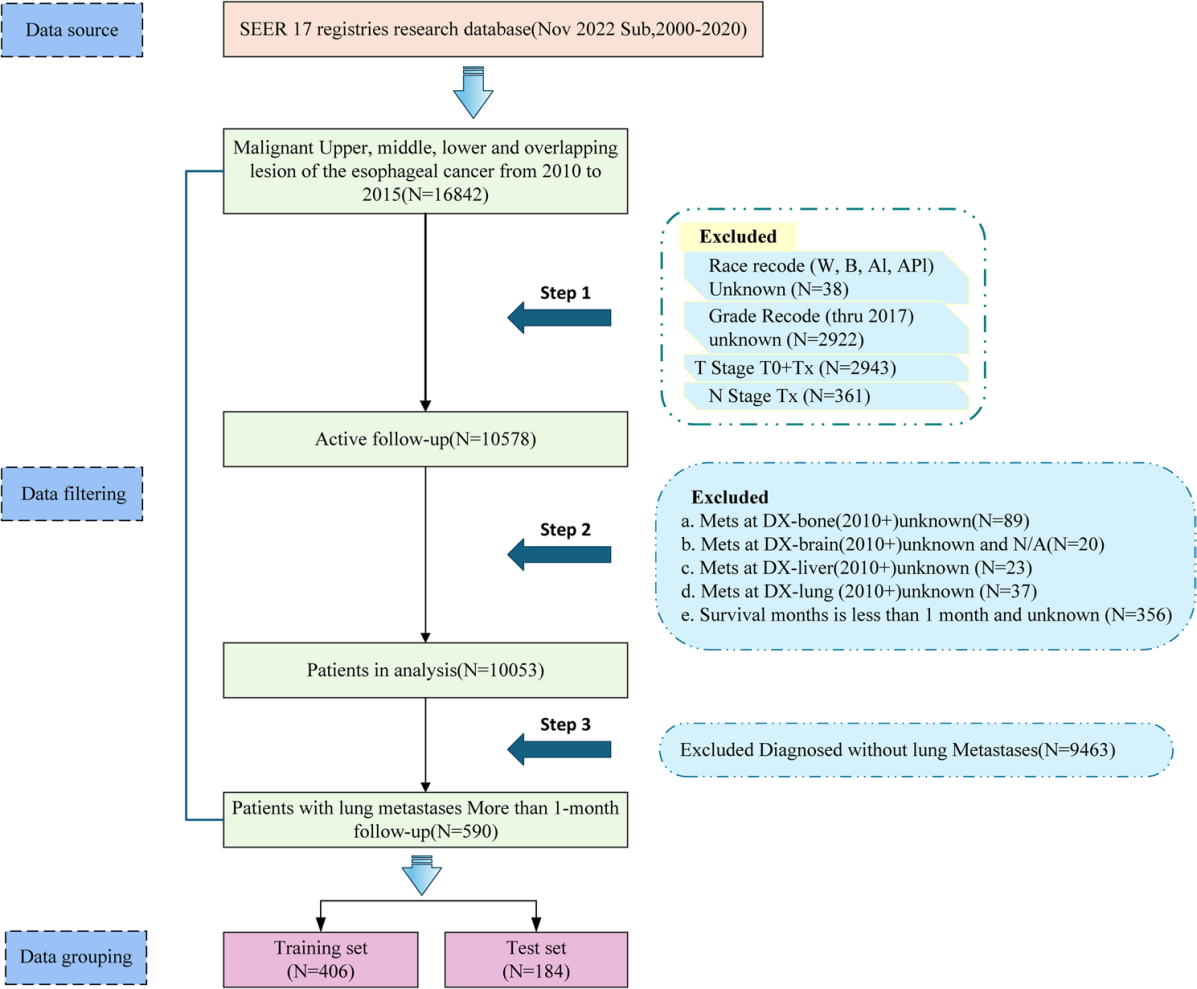
Sample screening process

The SEER database (https://seer.cancer.gov/) is a publicly accessible, authoritative cancer surveillance database system that is available to qualified researchers upon formal request. Data for this study was collected from the SEER*Stat software (version) from the SEER 17 registries research database (Nov 2022 Sub, 2000–2020). The SEER database started collecting data related to bone, brain, liver, and lung metastases from 2010, so this study included a total of 16,842 esophageal cancer patients from 2010 to 2015. The selection criteria were as follows: (1) patients with esophageal cancer, (2) primary sites were Upper third of esophagus (C15.3), Middle third of esophagus (C15.4), Lower third of esophagus (C15.5), and Overlapping lesion of esophagus (C15.8), (3) ICD-O-3 codes: 8052, 8070–8075, 8083, 8094, 8140, 8144, 8200, 8210–8211, 8244, 8255, 8260–8261, 8263, 8310, 8323, 8480–8481, 8574. Exclusion criteria were (1) unknown or incomplete follow-up data, (2) a follow-up time of at least one year, and (3) no occurrence of bone, brain, liver, or lung metastases (Fig. 1). The conduct and reporting of this study adhere to the TRIPOD guidelines.

## Clinical information retrieval

Data from the SEER database included 16 variables, including demographic information such as patient ID, year of diagnosis, age at diagnosis, gender, and race. Tumor-related variables included tumor location, histological type, T stage, N stage, and the presence of metastases (bone, brain, liver, lung). Survival follow-up variables included the number of months survived and the survival status of patients.

### Statistical analysis

After screening the SEER database for relevant data that met the inclusion and exclusion criteria, we first used the chi-square test or Wilcoxon rank-sum test to verify differences between variables associated with lung metastasis. Then, Kaplan-Meier survival curves were used to compare survival outcomes between patients with lung metastasis and those without lung metastasis. To identify risk factors for lung metastasis in esophageal cancer and construct a prognostic prediction model, the sample split command in R software was used to divide the data of 590 patients with lung metastasis from esophageal cancer into training and testing groups. Univariate and multivariable COX regression models were used on the training group to screen for important risk factors for lung metastasis in esophageal cancer. Based on the significant factors identified through multivariable COX regression, dynamic nomogram charts were constructed using R software to predict the probability of 1-year, 3-year, and 5-year overall survival (OS) in patients with esophageal cancer and lung metastasis.

### Model validation

To ensure the reliability and generalization ability of the constructed prognostic model, this study randomly divided the entire lung metastasis patient cohort into a training group and a validation group at a ratio of 7:3. The training group was used exclusively for model construction and parameter estimation, while the validation set was strictly used to evaluate the model’s generalization performance, thereby effectively avoiding overfitting and providing unbiased performance estimates. We primarily employed the C-index, ROC analysis, and calibration curves for model validation. We calculated the time-varying AUC values for predicting 1-year, 3-year, and 5-year survival rates to further assess the model’s predictive accuracy at different time points. The closer the AUC value is to 1, the higher the discriminative ability. Additionally, we generated calibration curves using the Bootstrap method to quantitatively assess the consistency between the model’s predicted survival probabilities and the actual observed survival probabilities. All analyses were performed using R software.

## Results

### Patient characteristics

After screening data on esophageal cancer patients from 2010 to 2015, this study included a total of 10,053 patient cases. As shown in Table 1, it is evident that there were significant differences in the baseline clinical characteristics between patients who experienced lung metastasis (590 cases) and those who did not (9,463 cases) (*P* < 0.05). Among the 590 esophageal cancer patients diagnosed with lung metastasis, individuals aged over 65 years were more commonly observed, constituting 52.5% of the cases. In terms of gender distribution, males accounted for a significantly larger proportion among lung metastasis patients, comprising 84.2%. The majority of the patients with lung metastasis were of Caucasian ethnicity, making up 79.3%, while African American patients represented 13.6%, and patients of other ethnicities constituted only 7.1%. Histological grading revealed that 41.7% of patients had Grade II tumors, 54.4% had Grade III tumors, and other grading categories made up less than 4% of cases. The primary tumor location in the lower third of the esophagus was predominant among most lung metastasis patients. Adenocarcinoma was the dominant histological type, accounting for a substantial 64.6%, significantly outnumbering squamous cell carcinoma cases. The distribution of T-stage showed the highest proportion of patients in T1 stage, the lowest in T2 stage, and a nearly equal distribution between T3 and T4 stages, at roughly a 1:1 ratio. More than 50% of the patients were classified as being in the N1 stage. Bone metastasis, brain metastasis, and liver metastasis comprised 20.3%, 4.6%, and 44.1% of patients, respectively.

**Table 1:** Clinical characteristics of 10,053 esophageal cancer patients

level	Without Lung M (*N* = 9463)	With Lung M (*N* = 590)	*P*-value
Age			
< 65	4009 (42.4)	280 (47.5)	0.017
>=65	5454 (57.6)	310 (52.5)	
Sex			
Male	7524 (79.5)	497 (84.2)	0.006
Female	1939 (20.5)	93 (15.8)	
Race			
White	8207 (86.7)	468 (79.3)	< 0.001
Black	774 (8.2)	80 (13.6)	
Others	482 (5.1)	42 (7.1)	
Grade			
I	710 (7.5)	20 (3.4)	< 0.001
II	4270 (45.1)	246 (41.7)	
III	4378 (46.3)	321 (54.4)	
IV	105 (1.1)	3 (0.5)	
Primary Site			
Upper third of esophagus	561 (5.9)	40 (6.8)	0.019
Middle third of esophagus	1649 (17.4)	112 (19.0)	
Lower third of esophagus	6860 (72.5)	400 (67.8)	
Overlapping lesion of esophagus	393 (4.2)	38 (6.4)	
Histology			
Squamous cell carcinoma	2911 (30.8)	209 (35.4)	0.02
Adenocarcinoma	6552 (69.2)	381 (64.6)	
T stage			
T1	2832 (29.9)	223 (37.8)	< 0.001
T2	1333 (14.1)	29 (4.9)	
T3	4299 (45.4)	163 (27.6)	
T4	999 (10.6)	175 (29.7)	
N stage			
N0	4127 (43.6)	147 (24.9)	< 0.001
N1	3908 (41.3)	347 (58.8)	
N2	1084 (11.5)	58 (9.8)	
N3	344 (3.6)	38 (6.4)	
Bone metastases			
NO	9082 (96.0)	470 (79.7)	< 0.001
YES	381 (4.0)	120 (20.3)	
brain metastases			
NO	9379 (99.1)	563 (95.4)	< 0.001
YES	84 (0.9)	27 (4.6)	
liver metastases			
NO	8727 (92.2)	330 (55.9)	< 0.001
YES	736 (7.8)	260 (44.1)	

### Risk factors for lung metastasis

Lung metastasis risk factors for esophageal cancer were analysed based on data from 10,053 patients with no missing information between 2010 and 2015. Results from the single-factor logistic analysis revealed that age, gender, ethnicity, histological grading, pathological type, T stage, N stage, bone metastasis, brain metastasis, and liver metastasis were significant influencing factors for the development of lung metastasis in esophageal cancer. Subsequently, these ten risk factors were incorporated into a multi-factor logistic model for estimation. The results in Table 2 showed that age, specifically being aged 65 years or older (vs. Age < 65; OR 1.13; 95% CI 0.94–1.36; *P* = 0.191), had no significant impact on the occurrence of lung metastasis in esophageal cancer. Female gender (vs. Male; OR 0.76; 95% CI 0.59–0.97; *P* = 0.028), T2 stage (vs. T1 stage; OR 0.32; 95% CI 0.21–0.48; *P* < 0.0001), T3 stage (vs. T1 stage; OR 0.47; 95% CI 0.37–0.59; *P* < 0.0001), adenocarcinoma histological type (vs. Squamous cell carcinoma; OR 0.66; 95% CI 0.48–0.75; *P* < 0.0001), and T4 stage (vs. T1; OR 1.36; 95% CI 1.07–0.72; *P* = 0.012) were all significantly negatively associated with the occurrence of lung metastasis at the time of esophageal cancer diagnosis. On the other hand, factors such as African American ethnicity (vs. White; OR 1.54; 95% CI 1.14–2.06; *P* = 0.005), other ethnicities (vs. White; OR 1.36; 95% CI 0.93–1.95; *P* = 0.097), Grade II histological grading (vs. Grade I; OR 1.76; 95% CI 1.11–2.96; *P* = 0.023), Grade III histological grading (vs. Grade I; OR 1.78; 95% CI 1.12–2.97; *P* = 0.020), T4 stage (vs. T1; OR 1.36; 95% CI 1.07–0.72; *P* = 0.012), bone metastases (vs. without bone metastases; OR 2.81; 95% CI 2.17–3.62; *P* < 0.0001), brain metastases (vs. without brain metastases; OR 2.65; 95% CI 1.57–4.36; *P* = 0.000), and liver metastases (vs. without liver metastases; OR 6.42; 95% CI 5.25–7.84; *P* < 0.0001) all exhibited a positive influence on the risk of lung metastasis.

**Table 2:** Single-factor and multi-factor logistic regression for analyzing the risk factors for developing lung metastases in esophageal cancer patients

Subject characteristics	single-factor OR (95% CI)	*P*-value	multi-factor OR (95% CI)	*P*-value
Age	0.81(0.69–0.96)	0.015	1.13(0.94–1.36)	0.191
Sex	0.73(0.58–0.91)	0.006	0.76(0.59–0.97)	0.028
Race				
Black	1.81(1.41–2.31)	0.000	1.54(1.14–2.06)	0.005
Others	1.53(1.09–2.1)	0.012	1.36(0.93–1.95)	0.097
Grade				
II	2.05(1.32–3.35)	0.002	1.76(1.11–2.96)	0.023
III	2.60(1.69–4.25)	< 0.0001	1.78(1.12–2.97)	0.020
IV	1.01(0.24–3.02)	0.982	0.90(0.20–2.79)	0.868
Primary Site				
Middle third of esophagus	0.95(0.66–1.40)			
Lower third of esophagus	0.82(0.59–1.16)			
Overlapping lesion of esophagus	1.36(0.85–2.16)			
Histology	0.81(0.68–0.97)	0.018	0.66(0.48–0.75)	< 0.0001
T stage				
T2	0.28(0.18–0.40)	< 0.0001	0.32(0.21–0.48)	< 0.0001
T3	0.48(0.39–0.59)	< 0.0001	0.47(0.37–0.59)	< 0.0001
T4	2.22(1.80–2.74)	< 0.0001	1.36(1.07–1.72)	0.012
N stage				
N1	2.49(2.05–3.05)	< 0.0001	2.10(1.69–2.62)	< 0.0001
N2	1.50(1.09–2.04)	0.010	1.77(1.21–2.41)	0.002
N3	3.10(2.11–4.46)	< 0.0001	2.76(1.79–4.16)	< 0.0001
Bone metastases	6.09(4.84–7.60)	< 0.0001	2.81(2.17–3.62)	< 0.0001
brain metastases	5.35(3.38–8.22)	< 0.0001	2.65(1.57–4.36)	0.000
liver metastases	9.34(7.81–11.17)	< 0.0001	6.42(5.25–7.84)	< 0.0001

### Survival and prognostic factors for esophageal cancer patients with lung metastases

**Fig. 2:**
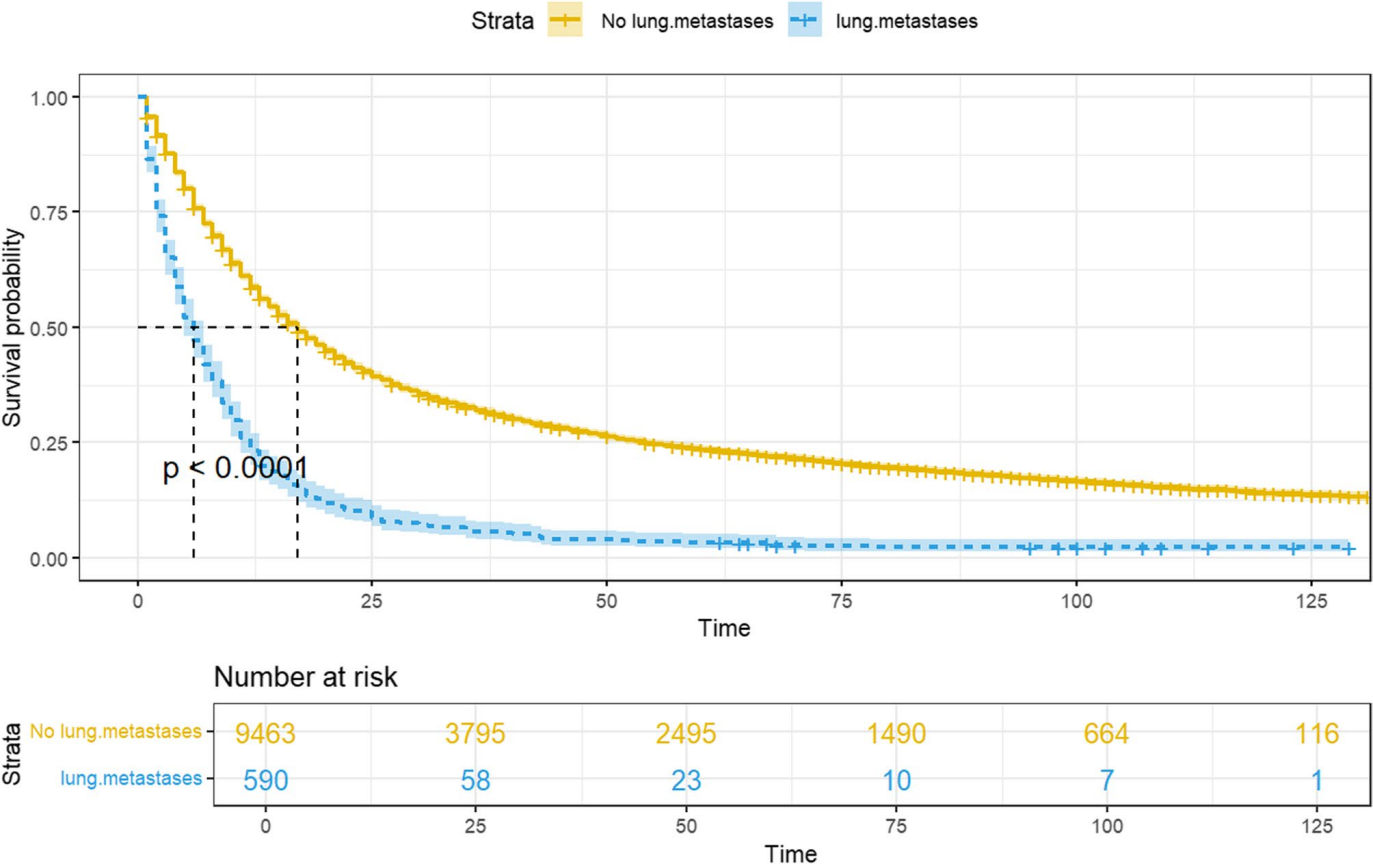
Survival curve for esophagus cancer patients with or without lung metastases

This study included a total of 590 esophageal cancer patients with lung metastasis from 2010 to 2015. The Kaplan-Meier survival curve in Fig. 2 shows that the median overall survival (OS) for people with esophageal cancer who got metastases in the lungs was 17 months (95% CI: 16–18), while the median OS for people without metastases in the lungs was 6 months (95% CI: 5–7). Furthermore, a significant difference in survival between the two groups was indicated by a p-value of less than 0.0001 obtained from the log-rank test.

To further explore the factors influencing the survival and prognosis of esophageal cancer with lung metastasis, the data were divided into train and test groups using the sample. split command in R. As shown in Table 3, the train group consisted of 406 patients, while the test group had 184 patients, maintaining a 7:3 ratio. In the train group, over half of the patients were aged 65 or older, with 18 more patients in this age group compared to those under 65. Male patients significantly outnumbered female patients, making up 83.5% of the group, while there were only 67 female patients. White individuals constituted 79.9% of the group, with Grade II and Grade III histological grading being the most prevalent, accounting for 96.3% of cases. 68.2% of patients with pulmonary metastases suffering from esophageal cancer had their primary tumor located in the lower third of the esophagus. Adenocarcinoma was the dominant histological type, representing 64%, almost twice the number of squamous cell carcinoma cases. Among the T stages, T2 was the least common in esophageal cancer patients with lung metastasis, with T1 and T4 stages being nearly equal in proportion. N1 stage patients made up 56.4% of the group. The percentage of patients with bone metastasis, brain metastasis, and liver metastasis was higher than that of patients without metastasis.

Single-factor COX analysis results indicated significant p-values for pathological grading, stages T, as well as metastases in the liver, brain, and bone. When these significant variables were further analyzed in a multivariable COX regression (as shown in Table 4), it confirmed that only Grade II (vs. Grade I; HR 2.03; 95% CI 1.09–3.77; *P* = 0.025), Grade III (vs. Grade I; HR 2.29; 95% CI 1.24–4.23; *P* = 0.008), T2 (vs. T1; HR 0.62; 95% CI 0.36–1.09; *P* = 0.099), T3 (vs. T1; HR 0.77; 95% CI 0.59-1.00; *P* = 0.050), and the presence of bone metastases (vs. without bone metastases; HR 1.47; 95% CI 1.14–1.89; *P* = 0.002) were factors influencing prognosis. Thus, the significant factors affecting the occurrence and prognosis of esophageal cancer with lung metastasis are pathological grading, T2 staging, T3 staging, and the existence of metastases in the bone.

**Fig. 3:**
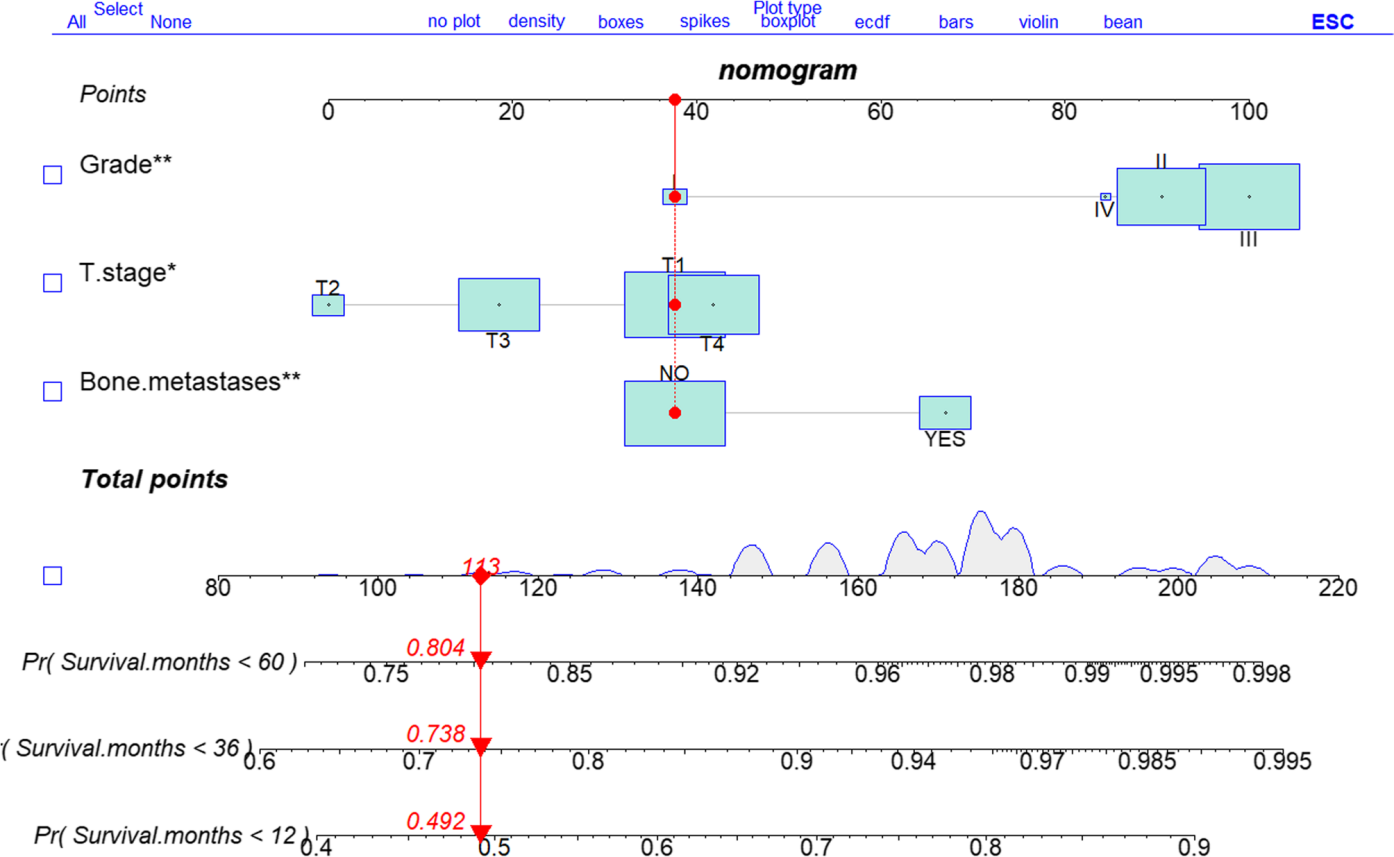
Dynamic nomogram for predicting OS in patients with lung metastases from esophageal cancer

**Fig. 4:**
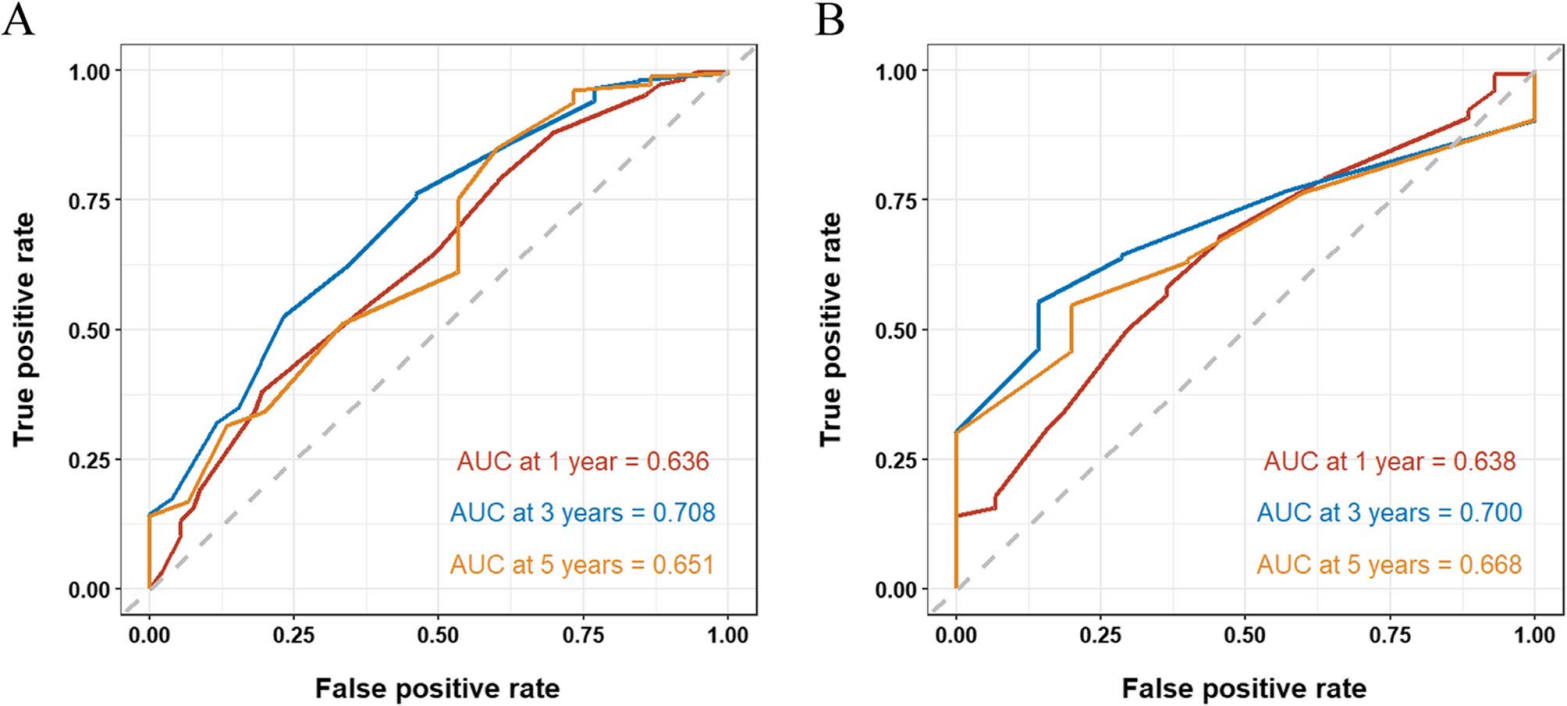
Train and teat group ROC curves for 1, 3, and 5 years. **A**: Training group; **B**: Test group

**Fig. 5:**
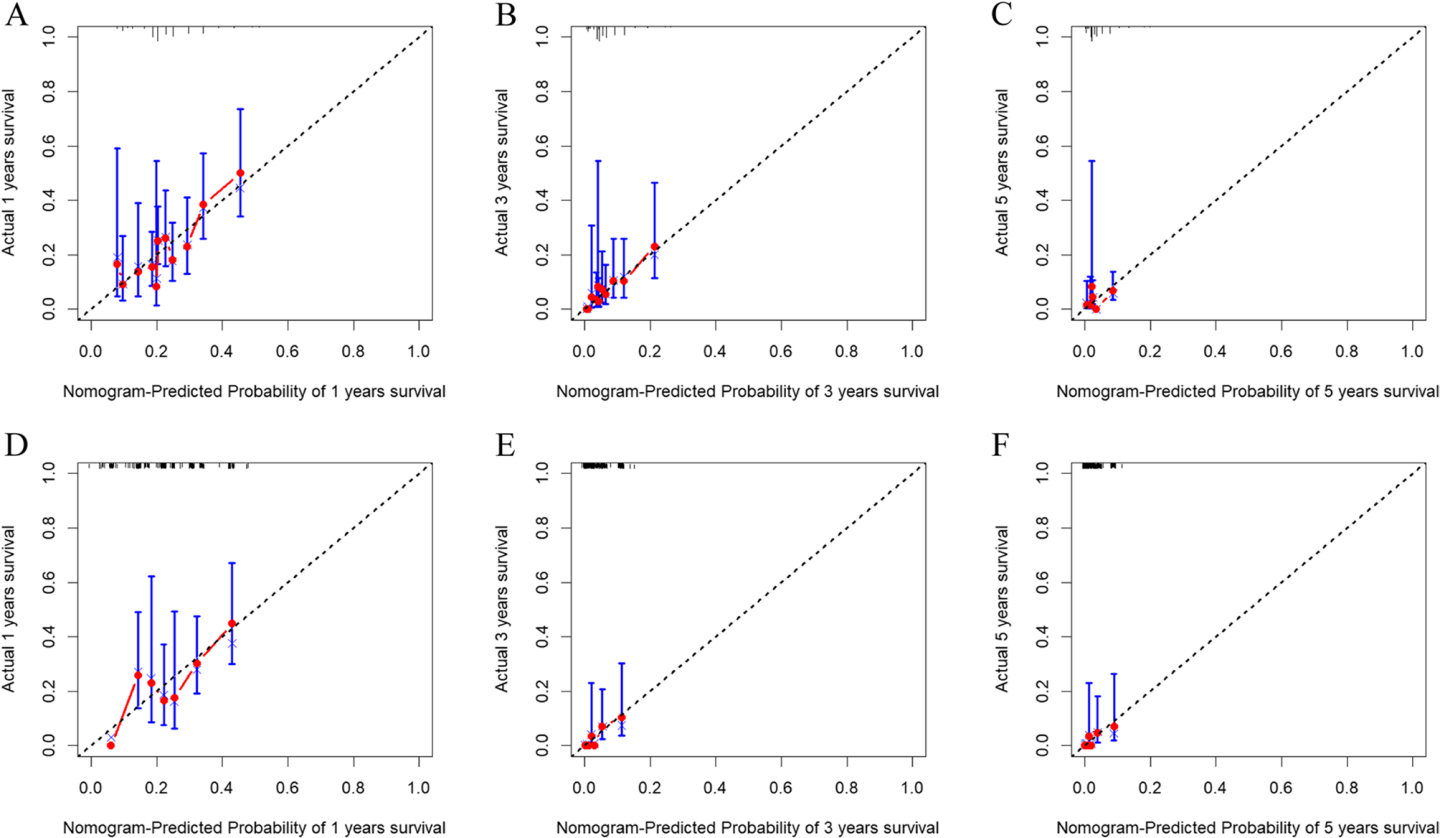
Train and test groups 1-year, 3-year, 5-year calibration curves. **A**-**C**: Training group; **D**-**F**: Test group

In this study, the authenticity of the predictive model was validated using calibration plots and calibration curves (as shown in Figs. 3, 4 and 5). Calibration plots (Fig. 3) showed the important factors from the multivariable COX analysis. The scores of three variables were added up to get an idea of the rates of survival for one, three, and five years. The predictive model exhibited a C-index value of 0.568(95% CI 0.535–0.601). The ROC analysis results of the training set (Fig. 4A) show that the AUC values for 1-year, 3-year, and 5-year risk prediction are 0.636, 0.708, and 0.651, respectively. The ROC analysis results of the validation set (Fig. 4B) show that the AUC values for 1-year, 3-year, and 5-year risk prediction are 0.638, 0.700, and 0.668, respectively. The calibration curves demonstrated that the internal validation accuracy for 1 year, 3 years, and 5 years predictions was excellent (Fig. 5).

**Table 3:** Clinical case characteristics of patients with lung metastasis of esophageal cancer in the train group and test group

level		Train(*N* = 406)		Test(*N* = 184)		*P*-value
Age						
< 65		194 (47.8)		86 (46.7)		
>=65		212 (52.2)		98 (53.3)		0.322
Sex						
Male		339 (83.5)		158 (85.9)		0.375
Female		67 (16.5)		26 (14.1)		
Race						
White		321 (79.1)		147 (79.9)		0.125
Black		58 (14.3)		22 (12.0)		
Others		27 (6.7)		15 (8.2)		
Grade						
I		13 (3.2)		7 (3.8)		0.033
II		171 (42.1)		75 (40.8)		
III		220 (54.2)		101 (54.9)		
IV		2 (0.5)		1 (0.5)		
Primary Site						
Upper third of esophagus		27 (6.7)		13 (7.1)		0.942
Middle third of esophagus		72 (17.7)		40 (21.7)		
Lower third of esophagus		277 (68.2)		123 (66.8)		
Overlapping lesion of esophagus		30 (7.4)		8 (4.3)		
Histology						
Squamous cell carcinoma		146 (36.0)		63 (34.2)		1.000
Adenocarcinoma		260 (64.0)		121 (65.8)		
T stage						
T1		159 (39.2)		64 (34.8)		0.605
T2		16 (3.9)		13 (7.1)		
T3		103 (25.4)		60 (32.6)		
T4		128 (31.5)		47 (25.5)		
N stage						
N0		110 (27.1)		37 (20.1)		0.780
N1		229 (56.4)		118 (64.1)		
N2		41 (10.1)		17 (9.2)		
N3		26 (6.4)		12 (6.5)		
Bone metastases						
NO		323 (79.6)		147 (79.9)		0.577
YES		83 (20.4)		37 (20.1)		
brain metastases						
NO		385 (94.8)		178 (96.7)		0.863
YES		21 (5.2)		6 (3.3)		
liver metastases						
NO		221 (54.4)		109 (59.2)		0.320
YES		185 (45.6)		75 (40.8)		

**Table 4:** Multi-factor Cox regression for analyzing the prognostic factors for esophageal cancer patients with lung metastases

Subject characteristics		Multi-factor HR (95% CI)	*P*-value
Grade			
I		1 (reference)	
II		2.03 (1.10–3.77)	0.025
III		2.29(1.24–4.23)	0.008
IV		1.84(0.40–8.43)	0.431
T stage			
T1		1 (reference)	
T2		0.62(0.36–1.09)	0.099
T3		0.77(0.59-1.00)	0.050
T4		1.08(0.85–1.37)	0.542
Bone metastases			
NO		1 (reference)	
YES		1.47(1.14–1.89)	0.003
brain metastases			
NO		1 (reference)	
YES		1.43(0.91–2.24)	0.117
liver metastases			
NO		1 (reference)	
YES		1.07(0.87–1.31)	0.516

## Discussion

Our study primarily focused on the survival and prognosis analysis of esophageal cancer with lung metastasis. The results showed that esophageal cancer patients with lung metastasis had a better prognosis than those without lung metastasis. Further analysis of risk factors for esophageal cancer patients with lung metastasis revealed that significant influencing factors included grade, T stage, and bone metastasis. In addition, we verified the accuracy of the model prediction through AUC curves and calibration curves. Existing studies on esophageal cancer metastasis are mostly based on limited clinical data and focus on exploring the effectiveness and improvement of specific treatment methods. These studies often lack the support of large amounts of data [20]– [21]. In this study, data was initially analyzed from 10,053 esophageal cancer patients between 2010 and 2015. The analysis of clinical characteristics, risk factors for lung metastasis, and prognostic factors specific to esophageal cancer patients with lung metastasis was performed. Age, T-stage, and pathology type were used as independent predictors of esophageal cancer lung metastasis in the study of Guo et al. [22], and in our study, we identified a number of factors increasing the risk of lung metastasis through multivariate logistic regression analysis, including male gender, grade III histologic grading, a higher T-stage, and the presence of bone, brain, and liver metastases [23]– [24]. However, factors such as age, race, and primary tumor location did not have a significant impact on lung metastasis. Additionally, patients with distant metastases in esophageal cancer have significantly worse prognosis and survival rates compared to those without metastases, with a 5-year survival rate of less than 5% [25–28]. Kaplan-Meier survival curves were employed to compare the survival rates of patients with and without pulmonary metastases in this investigation. The analysis results showed a significant difference in survival time, with patients without lung metastasis having a median survival time of more than 10 months longer. This underscores the impact of lung metastasis on the prognosis of esophageal cancer patients.

To further explore survival and prognostic factors, this study randomly divided data from 590 patients with esophageal cancer lung metastasis into a train group and a test group at a ratio of 7:3. Subsequently, the study conducted a detailed analysis and validation of prognostic factors for esophageal cancer lung metastasis. Ghazy’s research suggests that tumor differentiation is one of the key factors influencing the prognosis of metastatic esophageal cancer [29]. Our univariate and multivariate Cox regression analyses conducted by the train group confirmed that histological grade (specifically grade II and grade III), T2 stage, T3 stage, and the presence of bone metastasis are significant prognostic factors. Based on these factors, we constructed a nomogram model for predicting 1-year, 3-year, and 5-year overall survival rates. The model validation results show that the dynamic nomogram exhibits certain discriminatory performance at different time points. The AUC values for predicting 1-year, 3-year, and 5-year survival rates in the training set are 0.636, 0.708, and 0.651, respectively; in the validation set, the corresponding AUC values are 0.638, 0.700, and 0.668, respectively. Notably, the model demonstrated optimal and stable discriminatory performance in predicting 3-year survival rates (training set AUC = 0.708, validation set AUC = 0.700). Yu et al. believe that when the AUC value exceeds 0.7, the constructed prediction model is effective [30]. This result not only outperforms the prediction performance of other time points in the model, but also approaches or reaches the upper-middle level of similar prognostic models. The robustness of the model in the 3-year prediction window may stem from the higher event rate at this time point, which facilitates the model’s ability to capture more stable risk patterns. Although the AUC values for 1-year and 5-year predictions are relatively low, they remain above random guessing (AUC > 0.5), indicating basic discriminative efficacy. The limited performance of the 1-year prediction may be attributed to the significant influence of unaccounted-for non-traditional factors (such as postoperative complications and treatment tolerance) on short-term survival. The slight decline in the 5-year prediction performance may be associated with increased loss to follow-up, late non-cancer-related competing risks, and the evolution of tumor heterogeneity during long-term follow-up.

From a clinical translation perspective, this model demonstrates clear application potential in predicting 3-year survival and can be used as an auxiliary tool to identify patient groups with poor mid-term prognosis, enabling closer follow-up or more aggressive intervention strategies. The model is presented in the form of a dynamic nomogram, enhancing its clinical practicality and accessibility and facilitating individualized risk assessment.

## Conclusion

In summary, the results of this study offer significant insight into the risk and prognostic factors that are unique to esophageal cancer patients with pulmonary metastases. The model for prediction that was established in this research can assist clinicians in personalized treatment planning and better survival prediction for this particular group of patients.

However, this study has limitations, including the use of data only from the U.S., and the potential for data incompleteness and bias in the SEER database. Future research should focus on conducting larger-scale clinical studies to validate the results and investigate various treatment strategies to improve the survival rates and quality of life for esophageal cancer patients with lung metastasis. Future large-scale clinical research is needed to validate these results fully and explore various treatment strategies to enhance the survival and quality of life of esophageal cancer patients with lung metastasis.

## Data Availability

No datasets were generated or analysed during the current study.
